# Lifting the Concentration Limit of Mass Photometry by PEG Nanopatterning

**DOI:** 10.1021/acs.nanolett.4c01667

**Published:** 2024-07-01

**Authors:** Jiří Kratochvíl, Roi Asor, Seham Helmi, Weston B. Struwe, Philipp Kukura

**Affiliations:** † The Kavli Institute for Nanoscience Discovery, University of Oxford, Dorothy Crowfoot Hodgkin Building, South Parks Road, Oxford OX1 3QU, U.K.; ‡ Physical and Theoretical Chemistry Laboratory, Department of Chemistry, University of Oxford, South Parks Road, Oxford OX1 3QZ, U.K.; § Department of Biochemistry, University of Oxford, South Parks Road, Oxford OX1 3QU, U.K.

**Keywords:** mass photometry, surface passivation, nanoparticle lithography, protein−protein interactions

## Abstract

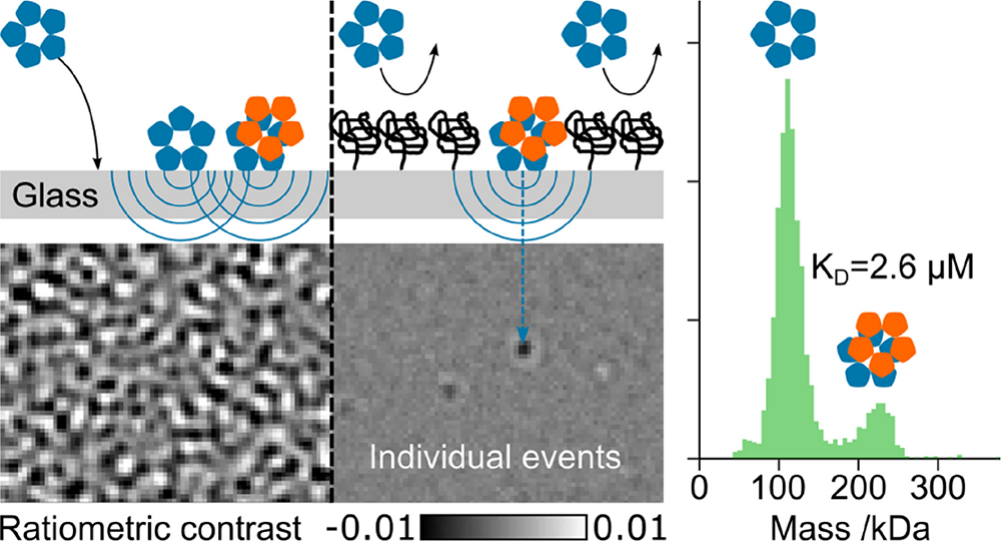

Mass photometry (MP) is a rapidly growing optical technique for label-free mass measurement of single biomolecules in solution. The underlying measurement principle provides numerous advantages over ensemble-based methods but has been limited to low analyte concentrations due to the need to uniquely and accurately quantify the binding of individual molecules to the measurement surface, which results in diffraction-limited spots. Here, we combine nanoparticle lithography with surface PEGylation to substantially lower surface binding, resulting in a 2 orders of magnitude improvement in the upper concentration limit associated with mass photometry. We demonstrate the facile tunability of degree of passivation, enabling measurements at increased analyte concentrations. These advances provide access to protein–protein interactions in the high nanomolar to low micromolar range, substantially expanding the application space of mass photometry.

Single-molecule mass measurement in solution by mass photometry (MP)^1^ has found broad applications across the life sciences, including studies of protein–protein interactions, characterization of structural heterogeneity, and biomolecular assembly.^2−8^ The measurement principle rests on detecting and imaging the interference of light scattered by the biomolecule with reference light provided by the reflection of a glass–water interface in the form of a microscope glass slide covered by an aqueous buffer (Figure 1a). Mass measurement, and in particular the differentiation of complexes and oligomers by mass, requires high measurement precision at the single-molecule level. This is usually achieved by integration for tens to hundreds of milliseconds to reduce shot-noise-induced fluctuations of the image background. As a result, individual molecules can be imaged with a high signal-to-noise ratio and their signal quantified.

**Figure 1:**
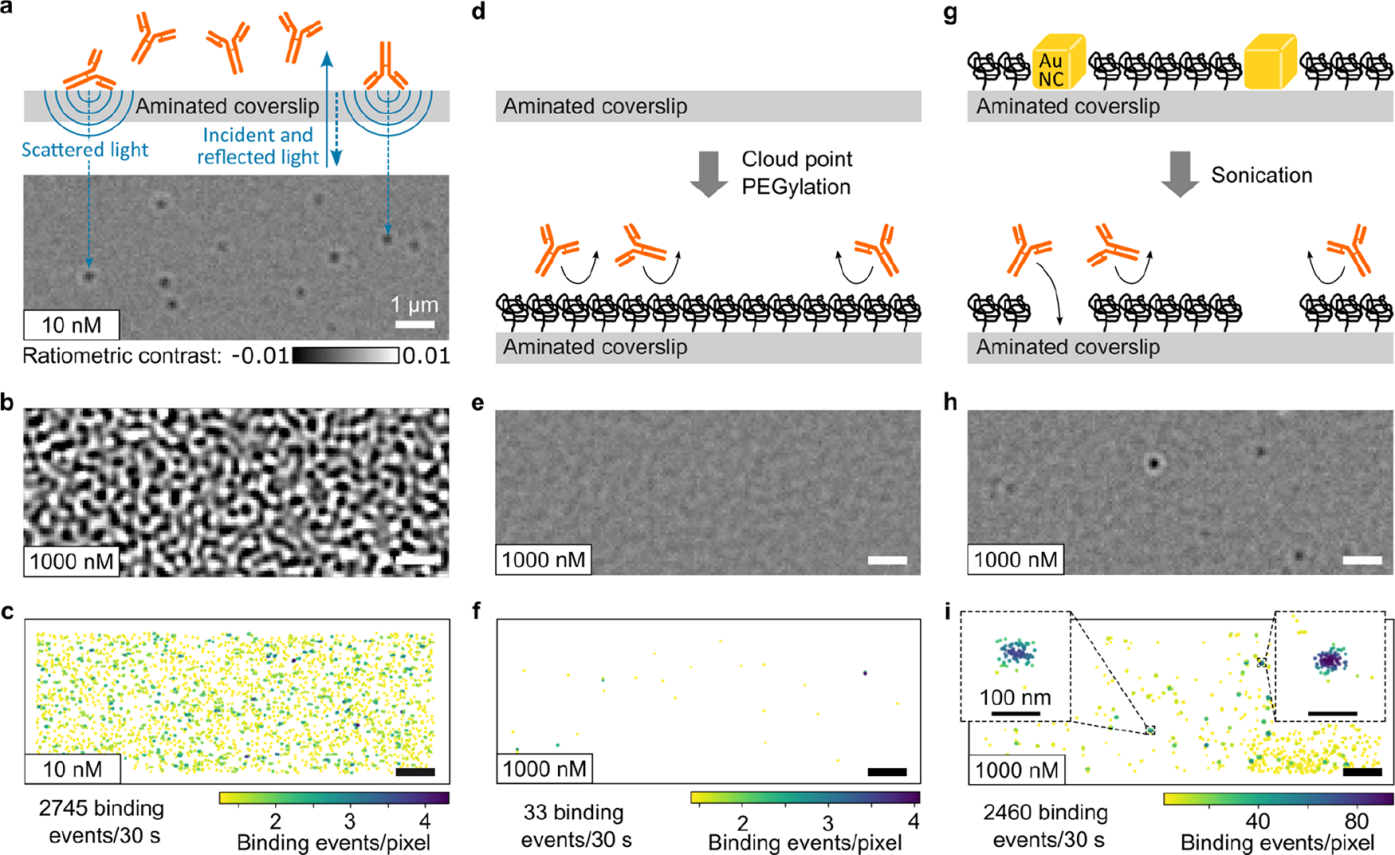
Nanocube lithography of passivated surfaces for high concentration mass photometry. (a) Principle of mass photometry including an MP image of 10 nM monoclonal SARS-CoV-2 spike antibody on a standard aminated microscope coverslip. (b) Equivalent snapshot at 1 μM analyte concentration. (c) Density map of landing events for a 30 s recording at 10 nM antibody concentration. (d) Schematic of high-density covalent PEGylation and the resulting passivation mechanism. (e) Corresponding MP image at a 1 μM antibody concentration. (f) Resulting landing density map. (g) Principle of nanoparticle-assisted nanopatterning of the PEGylated glass surface and mechanism of measurement on such a partially passivated surface. (h) Corresponding MP image at 1 μM antibody concentration. (i) Resulting landing density map including close-ups of regions with high landing density.

Precise measurement of the optical signal generated by individual molecules landing on the imaged surface, however, rests on their isolated observation. This requirement implies that no other landing events should take place in either the spatial or temporal vicinity of the event of interest, given the size of the diffraction-limited spot and integration time. As a result, most measurements performed by MP are limited to object concentrations on the order of a few tens of nM^2,9,10^ (Figure 1a), which prevents MP from investigating μM or weaker protein–protein interactions or operation at concentrations often used in cryo-electron microscopy and native mass spectrometry, because individual biomolecular landing events can no longer be distinguished (Figure 1b) in contrast to lower analyte concentrations (Figure 1c). Such concentration limitations are well-known in the optics-based single molecule community and have been addressed by approaches such as zero mode waveguides^11^ or rapid dilution.^12^ The former is incompatible with MP due to the associated optical heterogeneity of the detection surface. The latter represents an elegant solution but requires the use of microfluidics and cannot be performed at equilibrium.

An alternative approach to mitigate this limitation involves partial passivation of the glass surface, thereby reducing the frequency of landing events and enabling measurements at higher protein concentrations. The starting point needs to be a passivated surface that does not produce a signal in MP. As opposed to single-molecule fluorescence, reversible binding events with a residence time on the surface comparable to or longer than the temporal integration window produce a signal in MP.^13^ Passivation by BSA^14^ is generally suboptimal and produces an unwanted background from noncovalently attached and desorbing molecules. Lipid bilayers^15^ are challenging to pattern^16,17^ on the few tens of nm length scale due to their fluid character^18^ and increase the noise level during measurements.^19^ Steric repulsion by polymer brushes represents an alternative, such as those using zwitterionic materials,^20^ (DDS)-Tween-20,^21^ perfluoro-alkane brushes,^13^ or most commonly polyethylene glycol (PEG), also known as poly(ethylene oxide) (PEO).^22^ Attachment of PEG to the surface, so-called PEGylation, can be noncovalent^23^ or covalent.^24^ Increasing the PEG brush density by decreasing the hydrodynamic radius, known as cloud point PEGylation, is particularly advantageous due to the substantial associated improvement in passivation performance.^25−27^

Cloud point PEGylation of aminated coverslips (Figure 1d) results in outstanding passivation performance for MP, yielding only ∼1 landing event per second over the entire field of view (Figure 1e,f) even at concentrations where the corresponding bare glass surface is completely saturated (Figure 1b). The imaging background under these conditions is higher than that for measurements of low analyte concentrations in pure buffer due to rapidly diffusing species near the interface. While this slightly affects single molecule measurement precision, the associated increase is relatively minor, approximately doubling the mass-equivalent imaging background estimated as the standard deviation of ratiometric frames, for example, to 30 kDa from 15 kDa for an integration time of 28 ms (Figure 1e).

Having achieved robust surface passivation, we now need to partially reactivate the surface for biomolecular binding to enable MP measurements. Given that we require a substantial reduction of landing events to observe individual landing events to achieve accurate quantification, patterning on the micrometer scale is unsuitable because the landing density within the resulting patches would remain too high. We thus require many nonpassivated patches within the MP field of view that are smaller than the diffraction limit, which excludes the most popular polymer brush photopatterning methods.^28,29^ Such small patches sized on the <100 nm scale have been produced by STED lithography^30^ but with limited density. This limitation can be lifted by electron beam lithography^31^ but requires a conductive substrate, which is incompatible with standard MP. In principle, this could be overcome by scanning-based methods of patterning, e.g. by tip^32^ or focused ion beam patterning,^33^ which is, however, complex, and relatively low throughput. Given that EUV/X-ray lithography would be a suitable, but expensive candidate for larger-scale nanoscale patterning,^34−36^ we opted for a variation of nanoparticle lithography.^37−39^

In a first demonstration, we used gold nanocubes (AuNCs) to sparsely decorate an aminated glass coverslip before PEGylation (Figure 1g). We then remove the AuNCs by sonication, leaving the covalently attached PEG brushes intact. Performing an experiment at 1 μM analyte concentration (Figure 1h,i) results in a binding density reminiscent of that observed at 10 nM on a bare aminated glass surface (Figure 1c). A scatter plot of the landing event positions reveals clear signatures of areas of high landing density that resemble the area expected to be masked by AuNCs during the PEGylation procedure. These results suggest that nanoparticle-assisted lithography of PEG passivation enables MP measurements at roughly 2 orders of magnitude higher concentrations compared to aminated glass surfaces. Such reduction of the landing rate per 1 nM of analyte corresponds to the density of landed nanocubes and holds over a broad range of concentrations (Figure S1). We remark that aminated, positively charged glass surfaces maintain the universality of binding for proteins known from regular, negatively charged glass, likely thanks to a mix of positive and negative charges on the protein surface, ensuring sufficient interaction for adsorption. Nevertheless, in extreme cases, binding can be disrupted, for example, for nucleic acids on regular glass, as both are exclusively negatively charged in a neutral solution.

While these results obtained with AuNCs serve as a proof-of-concept, including super-resolution imaging of the AuNC-covered areas, tuning surface density is nontrivial with AuNCs due to the need for nanoparticle imprint lithography,^40^ surface modification,^41^ or functionalization^42^ to improve surface binding. Silica nanospheres (SNPs), on the other hand, naturally disperse in water and bind homogeneously at higher surface densities to aminated glass coverslips. In fact, this process can be observed in real-time using a mass photometer, starting with features arising from individual particles and concluding in a speckle pattern once the average interparticle distance becomes comparable to the diffraction limit (Figure 2a). The resulting PEGylated surfaces after nanoparticle removal exhibit an indistinguishable optical heterogeneity even for many nanoscopic PEG defects (Figure 2b). The associated scatter plot for biomolecular landing events now is evenly distributed across the field of view, as expected for a high density of nanoscopic defects that enable biomolecular binding (Figure 2c). Comparing the number of landing events on standard aminated glass and nanohole-PEG, we can estimate the ratio of hole to surface area to be on the order of 1:40. Similarly, comparing the drop of landing rates with a surface density of sparsely distributed 100 nm SNPs results in an estimate of the nanohole size of ∼32 nm (Figure S2).

**Figure 2:**
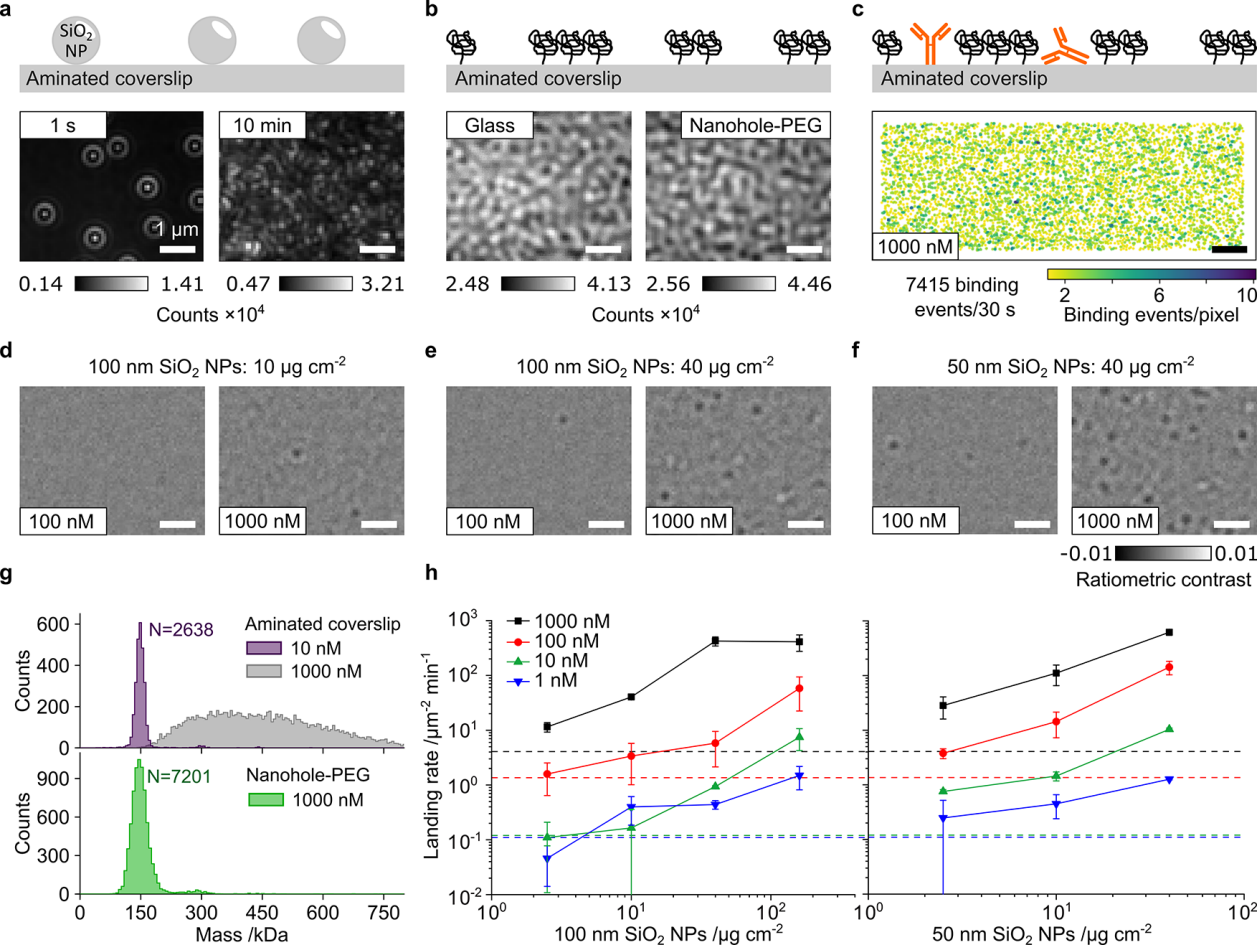
Passivation tunability with silica nanospheres (SNPs). (a) Schematic and corresponding MP images during 10 min of binding of 40 μg cm^–2^ of 100 nm SNPs. (b) Comparison of raw cover glass images with and without the nanopatterned PEG passivation layer. (c) Landing density map for antibodies at 1 μM concentration obtained with 40 μg cm^–2^ 100 nm SNPs. (d–f) MP images of antibody samples at 100 and 1000 nM concentrations using different SNP concentrations and sizes. (g) Comparison of antibody mass distributions on a standard aminated microscope coverslip and nanopatterned PEG obtained with 40 μg cm^–2^ 100 nm SNPs. (h) Resulting landing rates for silica nanospheres with 100 and 50 nm in diameter. The dashed lines indicate average landing rates for PEG without nanoholes; see Figure S1b. Coverslips were fabricated in triplicate; error bars indicate standard deviation based on at least 2 repeats for concentrations up to 10 nM or at least 3 repeats for higher concentrations.

The evolution of binding density with time (Figure 2a) and the availability of SNPs with different diameters mean that we can use both parameters to tune the total area and spatial distribution of the masked surface. Increasing nanoparticle concentration for the same particle size visibly increases the landing density of analyte (Figures 2d,e), as does halving the particle diameter for the same mass of SNPs used (Figure 2f). A surface prepared using 40 μg of 100 nm SNPs diluted in 200 μL to coat 1 cm^2^ enables MP measurement of antibodies at 1 μM concentration, producing roughly the same number of landing events as a standard coverslip at 10 nM, where the measurement fails completely at 1 μM (Figure 2g). To quantify the tunability and performance of this approach, we recorded landing rates as a function of analyte concentration, SNP diameter, and concentration; smaller 50 nm nanoparticles can be used to increase the landing rate further. In line with the increase in SNP surface coverage with concentration (Figure S2), we find up to 2 orders of magnitude tunability of landing rate with SNP concentration and size (Figure 2h). This enables selection of optimal surfaces as a function of desired analyte concentration, achieving landing rates ideally between 2 and 200 μm^–2^ min^–1^.

We can now compare MP performance in the presence and absence of nanostructured surfaces. In many cases, the motivation to work at higher analyte concentrations originates from a desire to capture interactions in the high nM to μM range. We begin with C-reactive protein, which is predominantly found in a pentameric form with a total mass of 115 kDa and forms decamers at higher concentrations.^43,44^ The maximum possible analyte concentration of 50 nM on standard aminated coverslips (Figure 3a) yields a single peak at 119 kDa (Figure 3b) and no significant signs of decamer formation.

**Figure 3:**
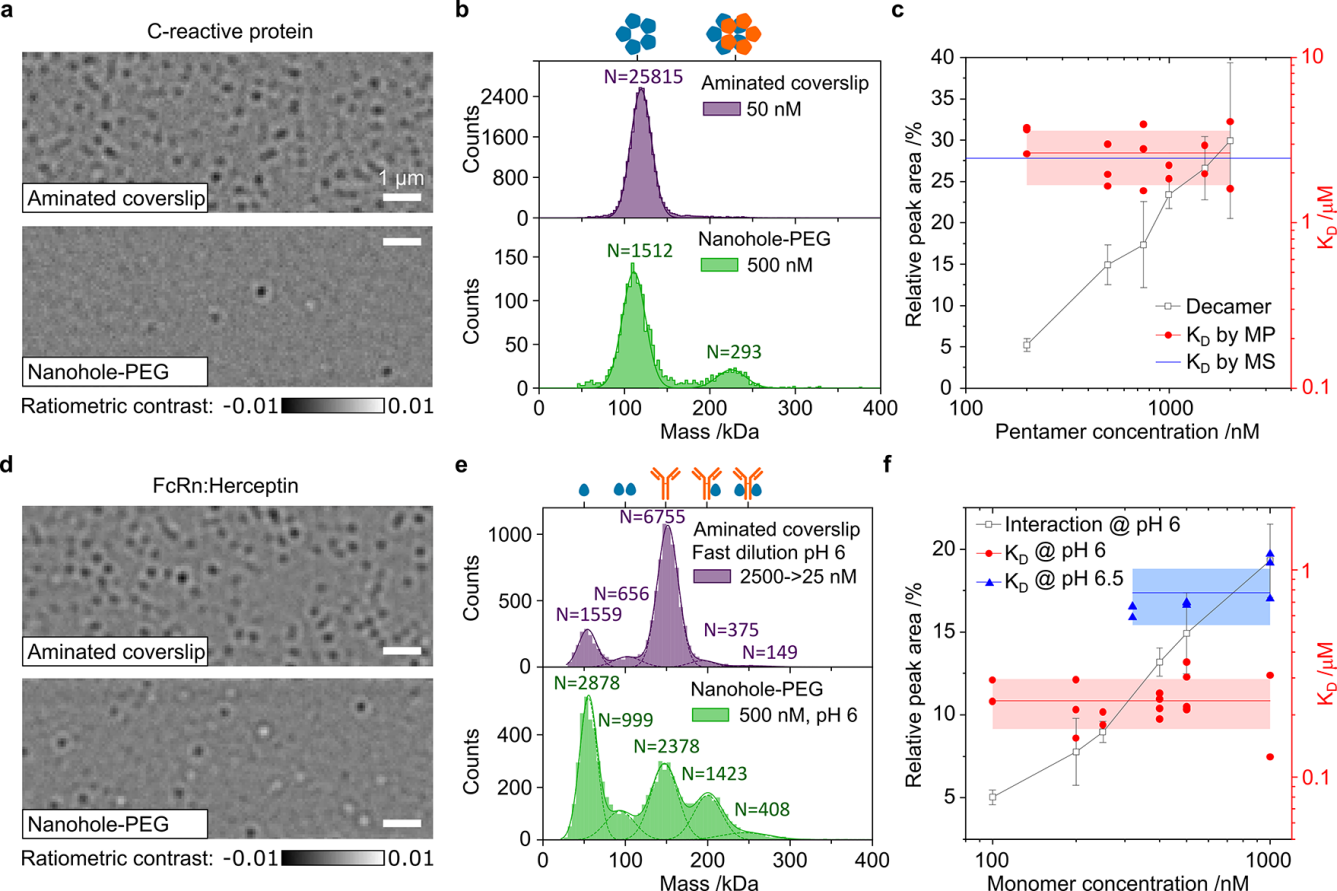
Nanopatterned PEG performance for high concentration MP. (a, b) Comparison of MP images and resulting mass histograms of C-reactive protein at 50 nM on standard cover glass and at 500 nM on nanopatterned PEG. (c) Resulting decamer fraction and associated *K*_D_ as a function of pentamer concentration. (d, e) Comparison of MP images and resulting mass histograms of 1:1 FcRn:Herceptin mixtures on standard cover glass and on nanopatterned PEG at indicated dilutions and pH. (f) Resulting complex fraction and *K*_D_ as a function of monomer concentration.

Repeating the measurement on a nanohole-PEG surface prepared using 10 μg cm^–2^ of 100 nm SNPs exhibits a much lower landing density even at 10× higher analyte concentration (Figure 3a). The corresponding mass histogram is still dominated by the pentameric form at 111 kDa, but it now exhibits a clear decamer signature at 225 kDa (Figure 3b). Given that we can quantify the number of both pentamers and decamers, we can readily deduce the associated affinity^3^ from individual spectra for dimerization as *K*_D_ = [monomer]^2^/[dimer], assuming that the measurement is taking place at equilibrium. Repeating this process across different analyte concentrations yields a *K*_D_ of 2.6 ± 0.9 μM. Repeating the experiment at 0.5, 1, and 7 μM concentrations with native mass spectrometry, performed as previously described,^45^ results in a similar *K*_D_ of 2.46 ± 0.14 μM; however, instead of PBS buffer, ammonium acetate is needed for desolvation with native mass spectrometry. MP experiments in ammonium acetate resulted in a *K*_D_ of 3.40 ± 1.23 μM, evidencing overall good agreement.

As a second application, we chose the interaction between the neonatal fragment crystallizable receptor (FcRn) and the therapeutic humanized IgG antibody trastuzumab (Herceptin) with molecular masses of 50 and 148 kDa, representative of a stereotypical protein:protein interaction. The chosen pair is particularly advantageous because the strength of the interaction varies with pH. This interaction was previously captured at low pH with MP using a rapid dilution method.^46^ Considering that fast dilution in the gasket can reveal the interaction,^47^ we 100× diluted an equimolar mixture of both proteins in the gasket to 25 nM. However, at pH 6, this still results in a high landing density (Figure 3d) and does not reveal strong evidence for FcRn:Herceptin complexes (Figure 3e), indicative of rapid complex dissociation. Repeating the experiment at 500 nM at equilibrium using a nanohole-PEG surface leads to a substantial reduction in the landing rate and a clear peak at the mass of the FcRn:Herceptin complex at 200 kDa. Using a similar procedure as that for C-reactive protein, we can estimate the affinity of this interaction as *K*_D_ = [FcRN][Herceptin]/[FcRN:Herceptin complex], which yields *K*_D_ = 0.23 ± 0.06 μM at pH 6.0 and *K*_D_ = 0.78 + 0.21 μM at pH 6.5 in good agreement with the literature.^48^

We have demonstrated a roughly 2 orders of magnitude improvement in the upper concentration limit for mass photometry by partially passivating microscope cover glass with nanopatterned, high-density PEG. This improvement is enabled by robust passivation of a high percentage of the glass surface, thereby substantially reducing the probability of successful immobilization of biomolecules diffusing in solution when they collide with the detection surface. Central to the success of the approach is creating a high density of unprotected areas with nanoscale dimensions separated by less than the diffraction limit. This approach maintains spatially uniform binding but at a much lower rate than that on standard cover glass. As a result, we continue to be able to identify and quantify individual landing events in time and space, which is critical for mass measurement precision and, thus, resolution. In addition to demonstrating a substantial drop in binding rate, we use these surfaces to visualize and characterize biomolecular interactions in the high nM and low μM range, such as the dimerization of C-reactive protein pentamers and the FcRn:IgG interaction as a function of pH.

Measurement at high concentration on a well-passivated surface increases the imaging background from diffusing and weakly bound species. This lowers the single molecule measurement precision when using standard analysis approaches, as evidenced by slightly increased peak widths, which results in an overall loss of mass resolution. In the future, this limitation can be overcome by a dedicated analysis pipeline aimed at averaging the fluctuating background or by varying the PEG chain length. In addition, restricting the analysis to clusters that clearly originate from deprotected areas will improve single molecule measurement precision and thus overall mass resolution. Nanoparticles used for nanohole fabrication, i.e., 100 and 50 nm, have a much larger diameter than the investigated proteins; however, surfaces with much smaller holes could be exploited for filtering out larger species, which would otherwise impair measurement. Similarly, larger nanoholes could be used to ensure facile binding of large biomolecular assemblies, if required.

While we cannot reach concentrations in the tens of μM range as in dilution-based methods^46^ due to the eventually overwhelming fluctuating background, our approach retains the simplicity of MP in terms of adding small amounts of sample directly and measurement at equilibrium, rather than requiring microfluidics or relying on long dissociation rates. Together with the high achievable dynamic range, the improvements presented here allow robust access to the few μM regime of biomolecular interactions and analyte concentrations, substantially expanding the application space of MP.

## Coverslip Amination

Coverslips were cleaned by sonication in acetone, 1:1 Milli-Q water:isopropanol solution, and Milli-Q, each for 5 min, followed by nitrogen blow-drying. Coverslips were then activated with hydroxyl groups in an oxygen plasma cleaner (Zepto-BRS 200, Diener electronic) for 8 min at 50% power. Plasma-activated coverslips were promptly inserted into a preheated solution of 2% (3-Aminopropyl)triethoxysilane (APTES, 99%, Sigma-Aldrich) in acetone (99%, HPLC grade). Coverslips were silanized at 40–50 °C for 1 h on a magnetic stirrer; during that time, the beaker with coverslips was sonicated for 1 min. The slides were subsequently twice sonicated in acetone for 5 and 1 min in Milli-Q water and blow dried with nitrogen.

## Nanoparticle Landing as a Nanoscale Mask

Immediately after surface amination, 1 × 1 cm square reusable gaskets (CultureWell, Grace Bio-Labs) were placed on coverslips and filled with 100 μL of Milli-Q water, and then 100 μL of Au nanocubes (5.2 pM, Nanopartz) or Milli-Q-diluted SiO_2_ nanospheres (AlphaNanotech) solution was pipetted under the water level. After 10 min of nanoparticle landing, the gasket was pipet-washed with Milli-Q, to remove remaining nanoparticles from solution. It is essential that drying of the surface with landed nanoparticles is avoided to prevent aggregation; therefore, during any liquid exchange, we always kept 50 μL of liquid in the gasket and changed the liquid by pipetting 450 μL in and out.

## Surface PEGylation and Nanoparticle Removal

Sodium carbonate/bicarbonate stock buffer at pH 9.5 was prepared by mixing sodium carbonate with bicarbonate at a mass ratio of 44:100. Then 1.044 g of potassium sulfate was added to 10 mL of buffer to prepare 0.6 M solution (PEG-buffer). At first, Milli-Q water in the gasket was replaced with the PEG-buffer, while the surface was kept wet. Then, the PEG-buffer was pipetted onto aliquoted mPEG-MW5000-SVA powder (Laysan Bio) to prepare 20% w/v PEG solution and promptly mixed by using a vortex. The resulting liquid is matte white (at 0.55 M potassium sulfate solution, the liquid is clear). A 50 μL portion of PEG solution was immediately transferred to the well filled with 50 μL of PEG buffer to obtain 10% w/v PEG in the gasket. Coverslips were incubated with PEG in the dark for 1 h at room temperature. After PEGylation, coverslips were twice sonicated in Milli-Q for 5 min with the gasket on and then nitrogen blow-dried and inserted into a 50 mL falcon tube with a silica gel bag or beads. The tube was filled with nitrogen, taped with parafilm, and immediately placed at −20 °C for long-term storage.

## Using Nanohole-PEG for Mass Photometry

Nanohole-PEG coverslips were removed from the freezer, then a gasket with four 3 mm wells (CultureWell, Grace Bio-Labs) was inserted into a large square gasket, and measurements were taken within 1 h using a mass photometer (TwoMP, Refeyn). The field of view was always moved to a nonpreviously exposed one and manually refocused just before starting the measurement to prevent photodegradation of the PEG passivation layer.

## Mass Photometry Measurements

Measurements of SARS-CoV-2 spike antibodies (Recombinant, Mouse mAb, Sino Biological) were performed in PBS buffer (DPBS, Gibco), human C-reactive protein (Sigma-Aldrich) oligomerization in PBS at pH 7.2 and the FcRn (recombinant human, C-His-Avi tag, host cell HEK293, Stratech) interaction with Herceptin (Trastuzumab, 600 mg/5 mL solution for injection, Roche) in PBS buffer at pH 6.0 and 6.5 (tailored by HCl addition). Fast manual dilution of the FcRn:Herceptin mixture was performed by placing a 49.5 μL droplet of buffer on a gasket and pipetting 0.5 μL of protein–antibody mixture into that droplet. The acquisition was initiated after mixing the well by pipetting up and down, which took less than 5 s. The contrast to mass calibration was performed as described previously.^19^ An integration time of 28 ms was used for movie processing, except for samples containing small FcRn proteins, where a longer integration time of 80 ms was needed to increase the signal-to-noise ratio. For measurement of nanoparticle landing, the laser power was reduced to avoid camera oversaturation by lowering the AOD offset to 1.5 V.

## Supporting Information Available

Quantitative performance of nanopatterned-PEG fabricated by nanocube lithography, including raw images of landed nanocubes and nanopatterned-PEG after nanocube removal, comparison of antibody titration on PEG, nanopatterned-PEG and glass coverslips, comparison of spectra acquired on glass and nanopatterned-PEG; Scaling of landing density with silica nanoparticle concentration and size, including raw and ratiometric frames and estimation of nanohole size. (PDF)
