# Supplementary material for: Lifting the Concentration Limit of Mass Photometry by PEG Nanopatterning
Source: Nano Lett. 2024 Jul 1;24(33):10032–9. doi: 10.1021/acs.nanolett.4c01667 (PMC11342371; doi:10.1021/acs.nanolett.4c01667)
Supplement: Supplementary file 1 — nl4c01667_si_001.pdf [file nl4c01667_si_001.pdf]

Lifting the Concentration Limit of Mass Photometry by PEG Nanopatterning

Jiří Kratochvíl,[1,2] Roi Asor,[1,2] Seham Helmi,[1,2] Weston B. Struwe,[1,3] and Philipp Kukura[1,2,]*

[1]The Kavli Institute for Nanoscience Discovery, University of Oxford, Dorothy Crowfoot Hodgkin Building, South Parks Road, Oxford OX1 3QU, U.K.

[2]Physical and Theoretical Chemistry Laboratory, Department of Chemistry, University of Oxford, South Parks Road, Oxford OX1 3QZ, U.K.

[3]Department of Biochemistry, University of Oxford, South Parks Road, Oxford OX1 3QU, U.K.

*Email: philipp.kukura@chem.ox.ac.uk.

Supporting Information S1: Nanocube-nanohole-PEG Performance

Comparison of the native image of sparsely distributed Au nanocubes that were used for surface masking and the native image of the surface after PEGylation and nanocube sonication, which is indistinguishable from glass (**Figure S1a**). Considering that 31 nanocubes sized 100 nm mask 0.31 μm^2, i.e. ~1% of the imaged surface area of 46.87 μm^2, we anticipate approximately 2 orders of magnitude decrease in landing rate for the same concentration, or a similar landing rate at 2 orders of magnitude higher concentration. This matches well with the results in **Figure S1b**, specifically with both the observed drop in landing rate of SARS-CoV-2 spike antibodies in the range of 1 – 100 nM for nanopatterned-PEG and the comparable landing rate on nanopatterned-PEG at 100 or 1000 nM, with 1 or 10 nM on glass. Counts on glass >50 nM are likely an underestimate due to the excessive event density.

The landing rate at 1000 nM concentration is 1.5 orders of magnitude higher for nanocube-nanohole-PEG compared to noise events for a PEGylated surface without nanopatterning. **Figure S1c** shows a comparison of mass spectra on glass and nanocube-nanohole-PEG. Mass spectra at 10 nM on glass show peaks at 148 kDa and -147 kDa; the latter is caused by antibodies unbinding from the glass surface. At 1000 nM, the field of view is overcrowded, which results in symmetric noise peaks at 494 and -489 kDa, so the mass of antibodies cannot be estimated. Nanopatterned-PEG significantly reduces the number of landing events and enables measurement of the antibody peak at 146 kDa, meanwhile the unbinding peak at -131 kDa is diminished, owing to the fact that the surface exposed by nanoholes is aminated.

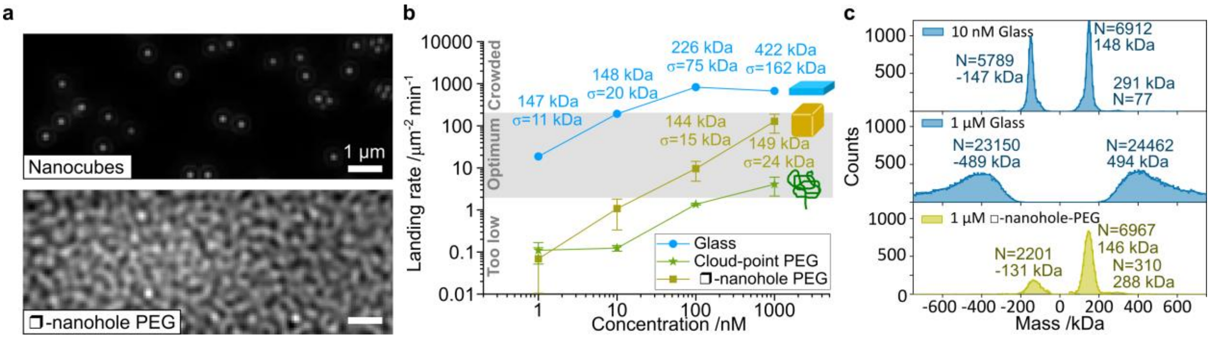

Figure S1: Quantitative performance of nanohole-PEG using sparse coverage by 100 nm Au nanocubes*. (a) Nanocubes on the surface before PEGylation and after PEGylation and their removal; images were normalised. (b) Comparison of landing rates of SARS-CoV-2 spike antibodies on glass, nanocube-nanohole-PEG and cloud-point PEG. Error bars indicate the standard deviation based on at least 2 repeats. (c) Comparison of mass spectra at concentrations of 10 and 1000 nM on glass and 1000 nM on nanopatterned-PEG.*

Supporting Information S2: Nanoparticles Landing

The landing of nanoparticles can be observed in raw images as diffraction-limited spots (**Figure S2)**. The low amount of 100 nm SNPs used for incubation with the sample resulted in a low surface density after 15 seconds of landing (**Figure S2a**). The same mass of 50 nm SNPs resulted in a much higher NP density because the weight of individual nanoparticles is 8x smaller (**Figure S2b**). A 16x larger amount of 100 nm SNPs increased the surface density, with SNPs almost indistinguishable from each other after 15 seconds (**Figure S2c**).

The resulting surface density using 2.5 µg cm^{-2} of 100 nm SNP can be estimated even after 10 minutes of landing as 1.26 μm^{-2}, which corresponds to one hole per 0.79 μm^2 (**Figure S2d**). Knowing the surface density of holes, we can estimate the hole size by comparing the landing rate on aminated glass and nanohole-PEG, which translates to an effective holes:total surface area ratio. The landing rate of 10 nM of antibodies on an aminated coverslip is 118.5 $\mu m^{-2}.min^{-1}$ (**Figure 1c**), and for 1 µM of antibodies on nanohole-PEG is 11.5 $\mu m^{-2}.min^{-1}$ (**Figure 2h**). When expressed per 1 nM of analyte concentration this converts to 11.85 $\mu m^{-2}.min^{-1}.nM^{-1}$ and 0.0115 $\mu m^{-2}.min^{-1}.nM^{-1}$ for aminated glass and nanohole-PEG, respectively, resulting in an estimate of holes:total surface ratio of 10^{-3}. Therefore, the hole area is about 0.79 x 10^{-3} μm^2, corresponding to a single-hole diameter of 32 nm, in good agreement with expectation based on the size of the SNP and PEG dimensions.

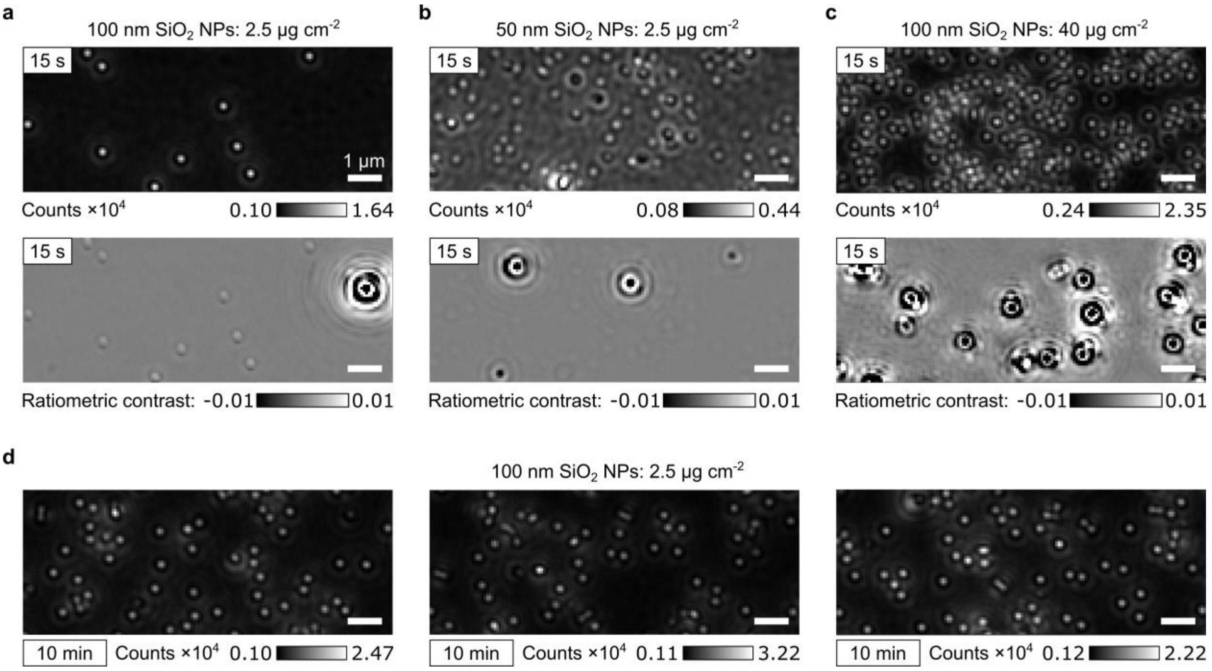

Figure S2: Raw and ratiometric frames of SNP landing for different concentrations and sizes. *Landing using (a) 2.5 µg cm^{-2} of 100 nm SNP, (b) 2.5 µg cm^{-2} of 50 nm SNP, and (c) 40 µg cm^{-2} of 100 nm SNP. (d) Raw image of final surface coverage using 2.5 µg cm^{-2} of 100 nm SNP at 3 random fields of view of area 46.87 μm^2 with 59 ± 3 particles on average. The averaging window for ratiometric frames was set to 100 ms.*
